# Supplementary material for: Integrated Analysis of Cortex Single-Cell Transcriptome and Serum Proteome Reveals the Novel Biomarkers in Alzheimer’s Disease
Source: Brain Sci. 2022 Aug 1;12(8):1022. doi: 10.3390/brainsci12081022 (PMC9405865; doi:10.3390/brainsci12081022)
Supplement: Supplementary file 1 [file brainsci-12-01022-s001.zip › supplementary Figures.pdf]

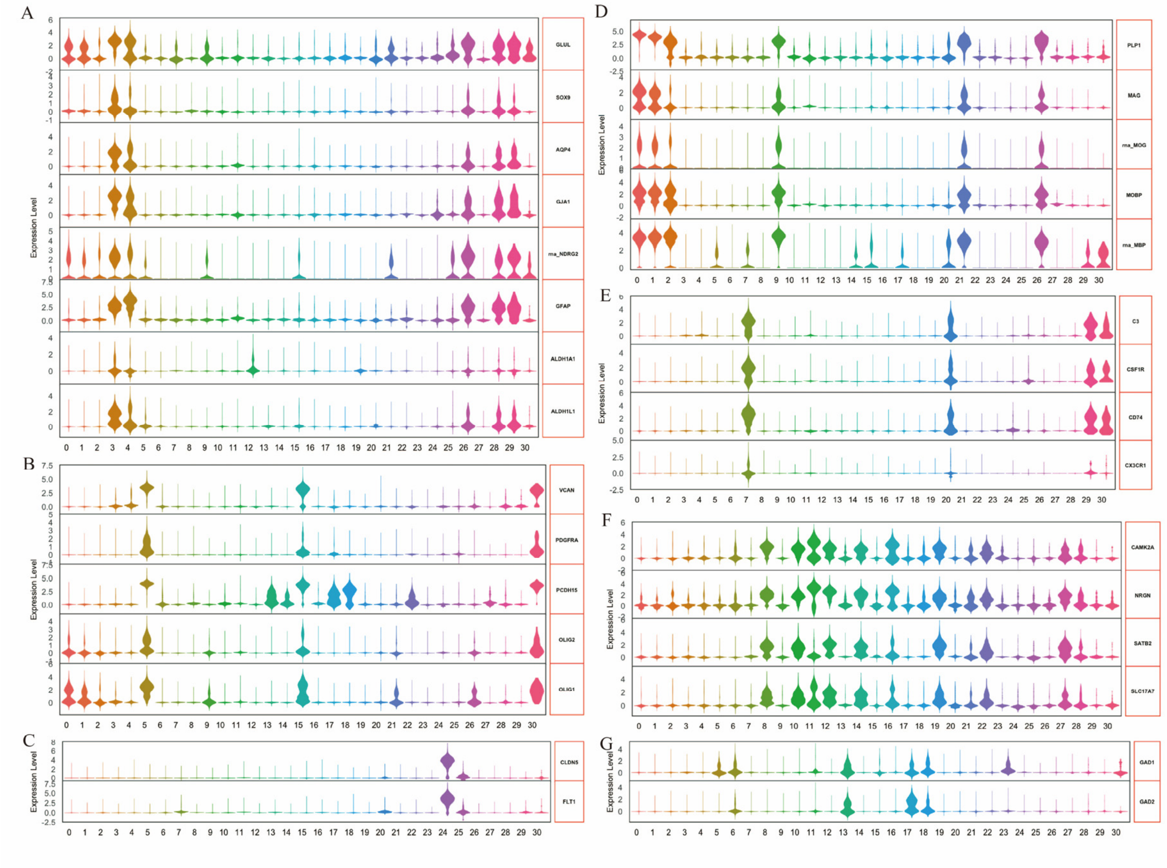

Figure S1. Expression levels of cell-type classical marker genes in all clusters.

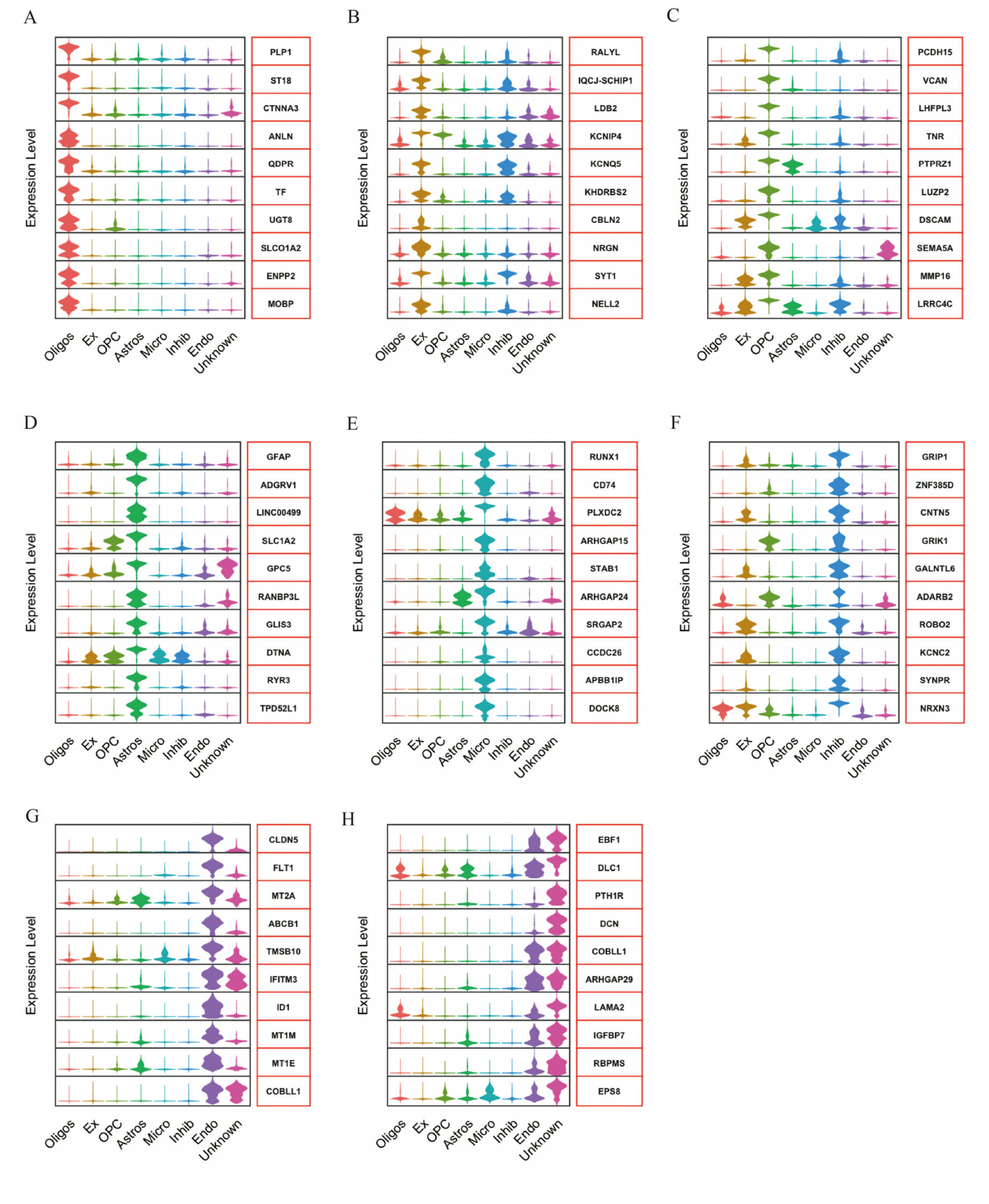

Figure S2. Specific differentially expressed genes expression levels of all cells in 8 cell types.
